# The Clinically-tested S1P Receptor Agonists, FTY720 and BAF312, Demonstrate Subtype-Specific Bradycardia (S1P_1_) and Hypertension (S1P_3_) in Rat

**DOI:** 10.1371/journal.pone.0052985

**Published:** 2012-12-28

**Authors:** Ryan M. Fryer, Akalushi Muthukumarana, Paul C. Harrison, Suzanne Nodop Mazurek, Rong Rhonda Chen, Kyle E. Harrington, Roger M. Dinallo, Joshua C. Horan, Lori Patnaude, Louise K. Modis, Glenn A. Reinhart

**Affiliations:** 1 Department of Cardiometabolic Disease Research, Boehringer-Ingelheim Pharmaceuticals Inc., Ridgefield, Connecticut, United States of America; 2 Department of Drug Discovery Support, Boehringer-Ingelheim Pharmaceuticals Inc., Ridgefield, Connecticut, United States of America; 3 Department of Medicinal Chemistry, Boehringer-Ingelheim Pharmaceuticals Inc., Ridgefield, Connecticut, United States of America; 4 Department of Immunology and Inflammation, Boehringer-Ingelheim Pharmaceuticals Inc., Ridgefield, Connecticut, United States of America; University of Illinois at Chicago, United States of America

## Abstract

Sphingosine-1-phospate (S1P) and S1P receptor agonists elicit mechanism-based effects on cardiovascular function *in vivo*. Indeed, FTY720 (non-selective S1P_X_ receptor agonist) produces modest hypertension in patients (2–3 mmHg in 1-yr trial) as well as acute bradycardia independent of changes in blood pressure. However, the precise receptor subtypes responsible is controversial, likely dependent upon the cardiovascular response in question (e.g. bradycardia, hypertension), and perhaps even species-dependent since functional differences in rodent, rabbit, and human have been suggested. Thus, we characterized the S1P receptor subtype specificity for each compound *in vitro* and, *in vivo*, the cardiovascular effects of FTY720 and the more selective S1P_1,5_ agonist, BAF312, were tested during acute i.v. infusion in anesthetized rats and after oral administration for 10 days in telemetry-instrumented conscious rats. Acute i.v. infusion of FTY720 (0.1, 0.3, 1.0 mg/kg/20 min) or BAF312 (0.5, 1.5, 5.0 mg/kg/20 min) elicited acute bradycardia in anesthetized rats demonstrating an S1P_1_ mediated mechanism-of-action. However, while FTY720 (0.5, 1.5, 5.0 mg/kg/d) elicited dose-dependent hypertension after multiple days of oral administration in rat at clinically relevant plasma concentrations (24-hr mean blood pressure = 8.4, 12.8, 16.2 mmHg above baseline *vs.* 3 mmHg in vehicle controls), BAF312 (0.3, 3.0, 30.0 mg/kg/d) had no significant effect on blood pressure at any dose tested suggesting that hypertension produced by FTY720 is mediated S1P_3_ receptors. In summary, in vitro selectivity results in combination with studies performed in anesthetized and conscious rats administered two clinically tested S1P agonists, FTY720 or BAF312, suggest that S1P_1_ receptors mediate bradycardia while hypertension is mediated by S1P_3_ receptor activation.

## Introduction

Sphingosine-1-phospate (S1P) and S1P receptor agonists elicit mechanism-based effects on cardiovascular function in animal models [1], [2] and in humans [3]. Indeed, S1P has been shown to regulate the contractility of a variety of vascular tissues, causing vasoconstriction in most vessels, and appears more important for contractile function of resistance vessels than of conduit vessels [4]. Other studies have demonstrated a mechanistic link between S1P and I_K(ACh)_ current in pacemaker cells [5]. However, the precise receptor subtypes (e.g. S1P_1_ and/or S1P_3_) responsible for these effects is controversial, dependent upon the cardiovascular effect in question (bradycardia, inotropy, or hypertension), and perhaps even dependent on species since functional differences in rodent, rabbit, and human have been suggested [6].

At least three of the S1P receptor subtypes (S1P_1,2,3_) are present in the heart and vasculature [7]–[9] although the predominant receptor subtype is dependent upon the specific cell type. The relative level of S1P_1_ exceeds that of S1P_2_ and S1P_3_ in the cardiac myocyte but S1P_3_ predominates in cardiac fibroblasts [8]. Moreover, the receptor subtypes responsible for specific cardiac or vascular functional effects has received increased attention in light of the discovery of novel agonists with putative receptor subtype selectivity [10]–[13] coupled with a desire to overcome cardiovascular liabilities observed in patients associated with the non-selective S1P receptor agonist, FTY720 [14].

Cellular studies have demonstrated that S1P receptor stimulation results in the activation of an inwardly rectifying acetylcholine-regulated K^+^ current, I_K(ACh)_, known to contribute to the cardiac resting membrane potential. Modulation of the current elicits changes in APD in multiple species [5], [15], including humans [16], that functionally results in both bradycardia and depression of contractile status of the heart *in vivo*. While S1P-mediated bradycardia is thought to be due to I_K(ACh)_ stimulation in SA nodal cells, independent of a central mechanism, the negative inotropic effects of S1P are thought to be a result of K_Ach_ activation in ventricular myocytes (as reviewed in [8], [17]). Similar to S1P receptor subtype expression in the heart, the specific receptor subtypes present in the vasculature are dependent on the resident cell type; S1P_1_ and S1P_3_ present in endothelial cells may contribute to NO-mediated vasodilation whereas S1P_2_ and S1P_3_ receptors in vascular smooth muscle cells have a role in Rho-mediated vasoconstriction (as reviewed in [7], [18]).

While FTY720 has been shown to elicit bradycardia and acute effects on vascular tone in pre-clinical animal models, it has not been adequately demonstrated that hypertension could have been detected in pre-clinical in vivo studies. In addition, it is unclear whether the mild degree of hypertension observed in patients is mediated by a classical renal mechanism or is instead vascular-based [19]. Finally the specific S1P subtype responsible for cardiovascular liabilities associated with receptor activation in rat has not yet been appropriately vetted in properly designed cardiovascular studies with clinically tested compounds of sufficient selectivity to investigate the mechanism. Thus, in the present study the effect of two differentially-selective S1P agonists, FTY720 and BAF312, that have been tested clinically and deemed clinically efficacious were investigated in anesthetized and telemetry-instrumented rats at clinically-relevant plasma concentrations to dissect the S1P receptor subtypes responsible for acute bradycardia and hypertension in vivo.

## Methods

All experiments were performed under protocols approved by the Boehringer-Ingelheim Institutional Animal Care and Use Committee and according to the United States Animal Welfare Act.

**Figure 1:**
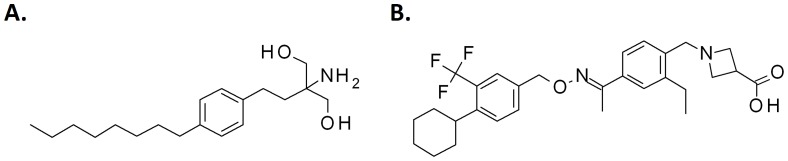
Chemical structure of FTY720 (A) and BAF312 (B), the latter as reported by Thompson Integrity and as referred to as BAF312 throughout the publication.

The chemical structure of BAF312 was recently reported by Novartis [13] and is shown in **Figure 1**. This compound was prepared as the zwitterion according to the synthetic procedures described [20] and crystallized according to procedures described in [21]. FTY720 hydrochloride was synthesized according to the procedure of [22]. (S)-FTY720-P was purchased from Toronto Research Chemicals Inc. The chemical structure of FTY720 is also shown in **Figure 1**.

### S1P Receptor Subtype Selectivity

Selectivity of compounds was confirmed with B arrestin recruitment assays with cells purchased from Life Technologies. Tango™ EDG1-bla U2OS cells (Human S1P1), Tango™ EDG3-bla U2OS cells (human S1P3), Tango™ EDG6-bla U2OS cells (human S1P4) and Tango™ EDG8-bla U2OS cells (human S1P5) cell lines were cultured and assayed according to manufacturers instructions with one modification; agonists incubated on the cells for 18 hours prior to addition of the GenBlazer substrate. Final DMSO concentration in the assay is 1%.

### Anesthetized Rat Cardiovascular Studies

The cardiovascular effects of FTY720 and BAF312 were tested in anesthetized rats (180–200 g). In brief, male Sprague Dawley rats (Charles River) were anesthetized with Inactin (110 mg/kg i.p.) and a tracheotomy performed. The femoral artery and veins were catheterized for measurement of mean arterial pressure and heart rate, collection of blood samples at 20-min intervals (0.2 mL/sample), and for compound infusion/hydration as previously described [23], [24]; blood samples were also withdrawn in vehicle-treated control rats. FTY720, infused as a hydrochloride salt (0.1, 0.3, 1.0 mg/kg/20-min free base equivalent solubilized in a 100% PEG-400 vehicle), or BAF312 (0.5, 1.5, 5.0 mg/kg/20-min solubilized in a 100% PEG-400 vehicle) were administered via three escalating 20-min infusions (0.011 mL/min) followed by a 1-hr post-treatment period. A blood sample was withdrawn at 20, 40, 60, and 120-minutes for analysis of plasma concentrations of the parent compound, and in the case of FTY720, also the phosphorylated metabolite, (S)-FTY720-P. Statistical significance (p<0.05) was based on a *t*-test comparing treatment change from baseline (baseline was defined as the period from −30 to 0 minutes) *vs.* vehicle controls change from baseline at each timepoint without adjustment for multiple comparisons. In a separate group of anesthetized rats in which left ventricular (LV) contractility was also recorded with a Millar pressure transducer introduced into the left ventricle, BAF312 was infused in the same dosing regimen as the studies described above (0.5, 1.5, 5.0 mg/kg/20-min solubilized in a 100% PEG-400 vehicle) and effects on LV dP/dt_max_ were recorded.

### Cardiovascular Profile in Telemetry-Instrumented Conscious Rats

Mean arterial pressure was assessed in conscious, freely moving male Sprague Dawley rats (n = 6–8/group; Charles River) instrumented with telemetry transmitters (DSI-Ponemah) and housed in metabolism cages as previously described [25], [26]. Tested compounds were solubilized in a 0.5% Methylcellulose/0.015% Tween 80 vehicle that was also dosed alone (p.o./QD, 5 mL/kg) as a control in the study. FTY720 was administered p.o. at 0.3, 1.0, 3.0, 10.0 mg/kg p.o. as a single dose or p.o./QD at 0.5, 1.5, and 5.0 mg/kg in a chronic study. BAF312 was administered p.o/QD at 0.3, 3.0, and 30 mg/kg as a single dose and in a chronic study. Mean arterial pressure and heart rate are shown as 1-hr and/or 24-hr mean values±SEM as described in the results. Blood samples in satellite rats were withdrawn at various timepoints as described in the results for analysis of plasma concentrations of the parent compound and, in the case of FTY720, also the phosphorylated metabolite, (S)-FTY720-P. Statistical analysis for the single-dose acute studies was based on one-way ANOVA to compare the treatment groups vs. vehicle for each time period after dosing (0–24 hrs, 24–48 hrs, and 48–72 hrs); statistical significance (p<0.05) was detected by Dunnett’s post-hoc test to compare treatment with vehicle controls. The statistical analysis in the chronic studies were based on one-way ANOVA with repeated measures to compare the treatment arms; statistical significance (p<0.05) was detected by Dunnett’s post-hoc test to compare treatment with vehicle controls.

## Results

BAF312 was confirmed to have the reported selectivity [13] of S1P_1_/_5_ agonism in vitro (B arrestin) with EC_50_s of 2.5/0.8 nM respectively and >10,000 fold selectivity over S1P_3_ and 100 fold selectivity over S1P_4_ **(** **Table 1** **)**. FTY720-P was run as a control and agonism on S1P_1,3,4,5_ with EC_50_ values of 0.4/54/26/1.8 nM were observed consistent with the selectivity profile previously reported [27]–[29].

**Table 1:** S1P receptor subtype selectivity of FTY720-P and BAF312, EC_50_ values reported in nM.

Compound	S1P_1_	S1P_3_	S1P_4_	S1P_5_
**FTY720-P**	0.4	54	26	1.8
**BAF312**	2.5	>10,000	245	0.8

**Figure 2:**
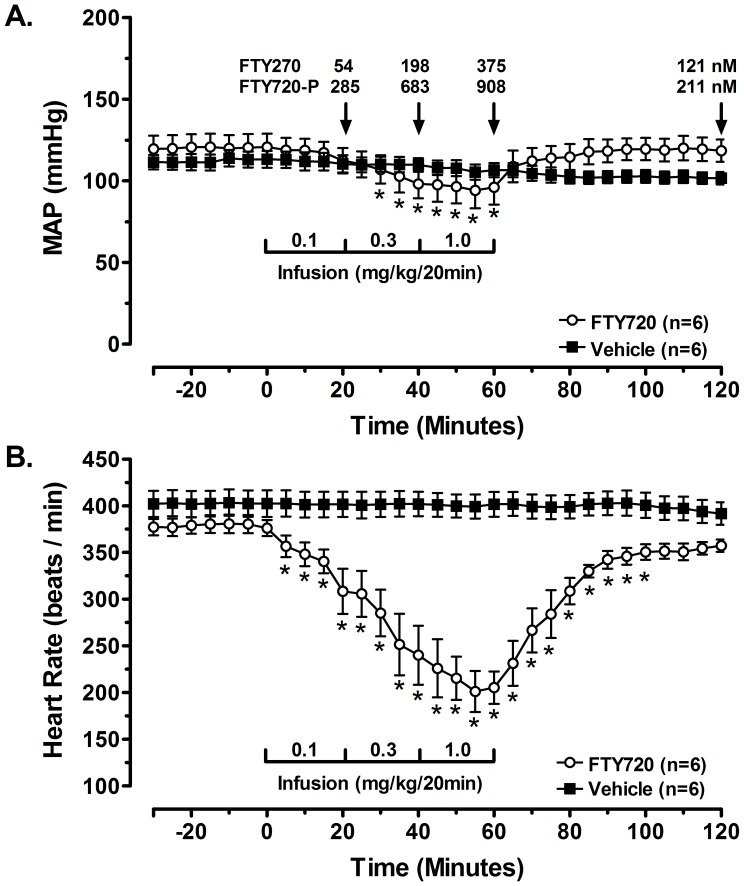
Effect of FTY720 on mean arterial pressure (MAP, Panel A) and heart rate (HR, Panel B) in anesthetized rats. Plasma concentrations at the end of each infusion period and at the end of the post-treatment period are shown in (A) in µM. At the end of each infusion period mean arterial pressure decreased to −9±3, −22±4, and −24±4 mmHg below baseline and heart rate decreased to −71±26, −139±37, and −173±26 beats/min below baseline values; *p<0.05 *vs.* the vehicle control group.

FTY720 was first profiled in anesthetized rats. In anesthetized rats plasma concentrations of parent and phosphorylated FTY720 at the end of each dosing period were 54±9, 198±13, and 375±46 nM (parent) and 285±87, 683±214, and 908±264 nM (phosphorylated), respectively; at the end of the 60-min post-treatment period values decreased to 121±5 nM (parent) and 211±17 nM (phosphorylated). At the end of each infusion period mean arterial pressure decreased to −9±3, −22±4, and −24±4 mmHg below baseline **(** **Figure 2** **, A)** and heart rate decreased to −71±26, −139±37, and −173±26 beats/min below baseline values **(** **Figure 2** **, B)**.

**Figure 3:**
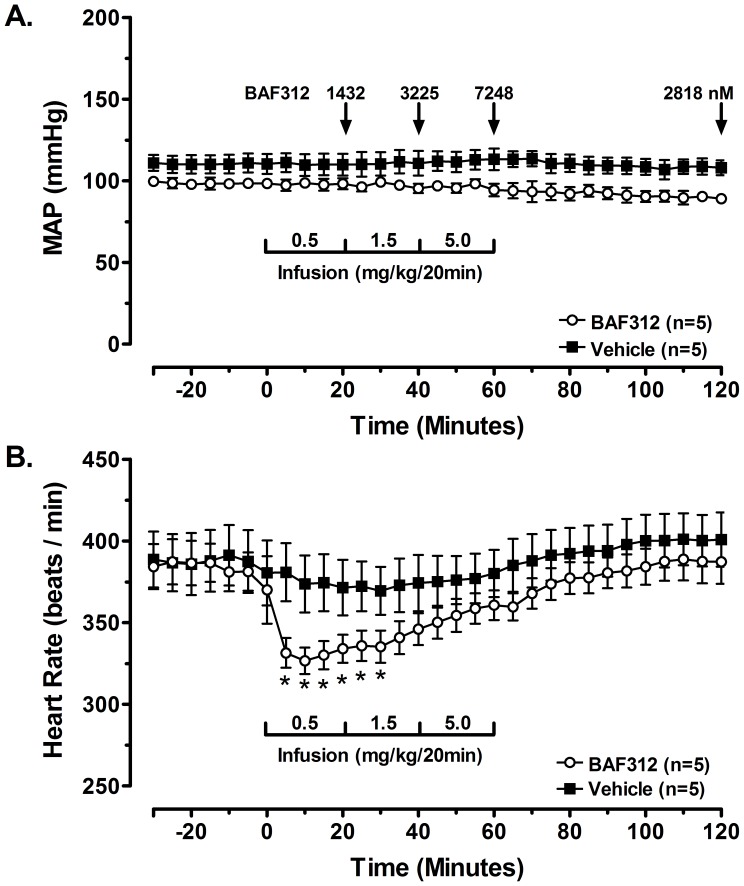
Effect of BAF312 on mean arterial pressure (MAP, Panel A) and heart rate (HR, Panel B) in anesthetized rats. Plasma concentrations at the end of each infusion period and at the end of the post-treatment period are shown in (A) in µM. BAF312 elicited an acute decrease in heart rate; 10-min into the first infusion period heart rate decreased to −51±8 beats/min (vehicle = −6±4 beats/min). However, despite an increase in plasma concentrations of BAF312 throughout the infusion period HR returned toward baseline and was not different from vehicle controls during the latter half of the 60-min infusion; *p<0.05 *vs.* the vehicle control group.

BAF312 was also profiled in the anesthetized rat. Plasma concentrations of BAF312 at the end of each dosing period were 1432±707, 3225±210, and 7248±517 nM, respectively; at the end of the 60-min post-treatment period values decreased to 2818±276 nM. BAF312 had no effect on mean arterial pressure **(** **Figure 3** **, A)**. Immediately upon infusion BAF312 elicited an acute decrease in heart rate; 10-min into the first infusion period heart rate decreased to −51±8 beats/min (vehicle = −6±4 beats/min) and values remained statistically reduced for 30-min **(** **Figure 3** **, B)**. However, despite an increase in plasma concentrations of BAF312 throughout the infusion period heart rate returned toward baseline and was not different from vehicle controls during the latter half of the 60-min infusion. In a separate group of animals instrumented with a Millar catheter into the left ventricle, BAF312 at the same doses elicited only a modest, but statistically-significant, decrease on LV dP/dt_max_ at the end of the 1.5 mg/kg i.v. infusion (to −5±4% below values in vehicle control animals; **Supplementary Figure, S1**).

**Figure 4:**
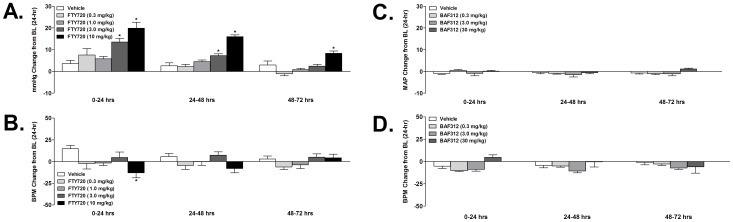
Effect of FTY720 (0.3, 1.0, 3.0, 10.0 mg/kg p.o.) on mean arterial pressure (A) and heart rate (B), and effect of BAF312 (0.3, 3.0, and 30 mg/kg p.o.) on mean arterial pressure (C) and heart rate (D), in conscious, telemetry-instrumented, rats after a single oral dose. Values are shown as 24-hr mean MAP (mmHg) and heart rate (beats/min, BPM) change from baseline. FTY720 elicited dose-dependent and sustained increases in MAP after a single oral dose, an effect that reached statistical significance in the 3 and 10 mg/kg dose groups (to 13.5±1.8 and 19.9±2.8 mmHg above baseline, respectively). Heart rate was acutely reduced (*vs.* vehicle) only in the 10 mg/kg group on day 1 (−13±6 beats/min). Thereafter, changes in heart rate were not different from vehicle control rats. BAF312 had no effect on MAP or HR at any dose tested in the study. Statistical analysis was performed on 24-hr mean values (*p<0.05 one-way ANOVA *vs.* vehicle with Dunnett’s post-test).

Prior to longer-term hypertension studies with FTY720 an acute, single oral-dose study was performed to define the lower and upper limits of a pharmacologically-active dose with respect to acute hemodynamic effects. Thus, FTY720 (0.3, 1.0, 3.0, and 10 mg/kg) was administered p.o. in telemetry instrumented rats as a single dose and hemodynamic values recorded for 72 hours post-treatment due to the prolonged half-life of the compound. Plasma concentrations of the parent at T_max_ were 15±3, 36±5, 91±19, and 301±31 nM, respectively; plasma concentrations of the phosphorylated metabolite at T_max_ were 83±3, 263±13, 360±55, and 1090±124 nM, respectively **(** **Table 2** **)**. Values of both the parent and metabolite were detectable through at least 72-hrs post-dose. Tissue concentrations in the brain, skeletal muscle, and CSF were also determined in a separate group of satellite rats (please see **Supplementary Figure, S2**). Relative to the vehicle control group, FTY720 elicited dose-dependent and sustained increases in mean arterial pressure after a single oral dose, an effect that reached statistical significance in the 3 and 10 mg/kg dose groups (to 13.5±1.8 and 19.9±2.8 mmHg above baseline, respectively, based on a 24-hr mean values on day 1 *vs.* 3.6±1.5 mmHg in vehicle controls, **Figure 4** **, A**). In the same study heart rate was acutely reduced (*vs.* vehicle) only in the 10 mg/kg group on day 1 (−13±6 beats/min *vs.* 15±3 beats/min in vehicle controls; **Figure 4** **, B**). Thereafter, changes in heart rate were not different from vehicle control rats. The acute hemodynamic effects of a single oral dose of BAF312 (0.3, 3.0, and 30 mg/kg) were also quantified. Compared to vehicle controls, the compound had no statistically significant effect on either mean arterial pressure **(** **Figure 4** **, C)** or heart rate **(** **Figure 4** **, D)** at all doses tested up to 30 mg/kg.

**Table 2:** Plasma concentrations of FTY720 and the phosphorylated form of FTY720, (S)-FTY720-P, after a single oral dose (0.3, 1.0, 3.0, 10.0 mg/kg) at 4, 8, 24, 48, and 72 hrs post-dose; values quantified by MS and are shown in nM (mean±SEM).

	FTY720				Phosphorylated FTY720			
Time	0.3	1.0	3.0	10.0	0.3	1.0	3.0	10.0
**4 hr**	10±5	25±3	62±4	146±18	66±3	172±16	285±29	559±159
**8 hr**	14±5	36±5	79±17	274±46	83±3	263±13	339±36	1145±505
**24 hr**	15±3	22±2	91±19	301±31	43±7	113±2	360±55	1090±124
**48 hr**	13±1	12±1	53±15	143±16	29±3	81±11	152±14	455±57
**72 hr**	7±2	10±1	34±4	101±21	4±4	26±4	165±2	629±157

Values in brain, muscle, and CSF at each timepoint in satellite rats in the 3 and 10 mg/kg dose groups are shown in the supplementary data.

We sought to ensure that effects of FTY720 on blood pressure could be tested in the absence of significant changes in heart rate and thus the doses selected were below that shown to elicit acute bradycardia after a single dose (observed only in the 10 mg/kg/d group in the single-dose study). In the repeat-dosing study with FTY720 (0.5, 1.5, and 5.0 mg/kg/d), plasma concentrations were also determined **(** **Table 3** **)**. On day 1, plasma concentrations of FTY720 at T_max_ in satellite rats (n = 3/group) were 13±2, 53±9, and 120±17 nM, respectively. Plasma concentrations of the phosphorylated metabolite at T_max_ were 103±24, 275±55, and 773±55 nM, respectively. Terminal plasma concentrations of FTY720 at T_max_ were 63±37, 81±4, and 229±21 nM, respectively, and concentrations of the phosphorylated metabolite at T_max_ were 546±358, 800±77, and 2913±92 nM, respectively. Concentrations of FTY720 and FTY720-P in heart at the end of the study were also determined, mean FTY720 tissue concentrations were 379, 490, and 1848 ng/g, respectively, in the 0.5, 1.5, and 5.0 mg/kg/d dose groups and corresponding concentrations of (S)-FTY720-P were 513, 757, and 3799 ng/g.

**Table 3:** Plasma (nM) concentrations of FTY720 and the phosphorylated form of FTY720 after oral administration (0.5, 1.5, 5.0 mg/kg/d) or BAF312 after oral administration (0.3, 3.0, 30.0 mg/kg/d) at 4, 8, and 24 hrs post-dose on Day 1 and at 0, 4, 8, and 24 hrs post-dose on the last day of the study; values were quantified by mass spectrometry and are shown as mean±SEM.

		FTY720			Phosphorylated FTY720			BAF312		
Day	Time	0.5	1.5	5.0	0.5	1.5	5.0	0.3	3.0	30.0
**Day 1**	**4 hr**	9±1	45±4	98±14	46±5	231±1	506±74	91±7	685±113	6395±390
	**8 hr**	13±2	53±9	102±4	103±24	275±55	696±279	65±8	584±81	4819±617
	**24 hr**	8±1	34±4	17±40	65±23	264±79	773±55	0±0	103±9	721±211
**Terminal**	**0-hr**	18±2	58±7	207±27	141±31	690±136	2344±356	nt	nt	nt
	**4 hr**	24±3	60±6	229±21	229±40	800±77	2913±238	81±8	735±73	8328±650
	**8 hr**	63±37	81±4	176±37	478±303	647±121	1508±316	54±7	480±33	5281±443
	**24 hr**	40±27	44±5	134±21	546±359	560±70	1561±230	13±6	98±8	942±117

Plasma concentrations in the repeat-dosing study with BAF312 (0.3, 3.0, and 30 mg/kg/d) were determined **(** **Table 3** **)**. On day 1, plasma concentrations of BAF312 at T_max_ (4 hrs) in satellite rats (n = 3/group) were 91±7, 685±113, and 6395±390 nM, respectively. Terminal plasma concentrations at T_max_ were 81±8, 735±73, and 8328±650 nM, respectively. Cardiac tissue concentrations of BAF312 were not determined.

**Figure 5:**
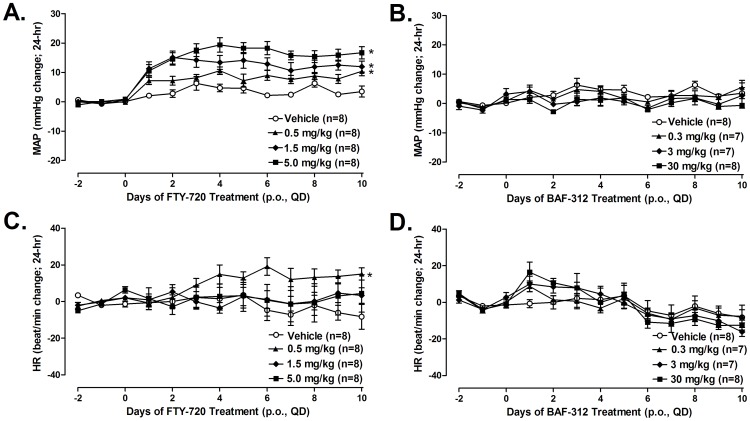
Effect of FTY720 and BAF312 on mean arterial pressure (MAP) and heart rate (HR) in conscious, telemetry-instrumented, rats during 10 days of daily oral administration. At doses that elicited no significant bradycardia, FTY720 elicited dose-dependent hypertension whereas BAF312 had no affect on MAP values at any dose tested during the study. FTY720 elicited dose-dependent increases in MAP at all doses tested (0.5, 1.5, and 5.0 mg/kg/d); 24-hr mean values over the treatment period increased 8.4±0.4, 12.8±0.4, and 16.2±0.8 mmHg, respectively, (vehicle = 3.7±0.5 mmHg) and values reached statistical significance in all treated groups (*p<0.05 *vs.* vehicle). However, BAF312 elicited no significant increase in mean arterial pressure during the study; average changes in 24-mean values during 14-days of treatment in the 0.3, 3.0, and 30 mg/kg/d dose groups were 3.0±0.5, 1.1±0.5, and 0.1±0.5 mmHg, respectively.

In the repeat-dosing study no differences in baseline mean arterial pressure were detected at any timepoint between the treatment groups. FTY720 (n = 8/group) elicited dose-dependent increases in mean arterial pressure at all doses tested (0.5, 1.5, and 5.0 mg/kg/d). Relative to baseline, the average mean arterial pressure values over the treatment period increased 8.4±0.4, 12.8±0.4, and 16.2±0.8 mmHg, respectively, (vehicle = 3.7±0.5 mmHg) and values reached statistical significance in all treated groups **(** **Figure 5, A** **)**. No differences in baseline mean arterial pressure between the groups were detected in the BAF312 arm of the study. BAF312 elicited no significant increase in mean arterial pressure during the study; average changes in 24-mean values during 14-days of treatment in the 0.3, 3.0, and 30 mg/kg/d dose groups were 3.0±0.5, 1.1±0.5, and 0.1±0.5 mmHg, respectively **(** **Figure 5, B** **)**. In the FTY720 arm of the study, the compound elicited no acute or sustained bradycardia at any dose tested **(** **Figure 5, C** **)**. Unexpectedly, values in the 1.5 mg/kg treatment group tended to be higher than those in all of the comparator groups, including higher doses of the compound (1.5 and 5.0 mg/kg) and those in the vehicle control group); 24-hr mean changes in heart rate in the vehicle, 0.5, 1.5, and 5.0 mg/kg dose groups were −2±1, 11±2, 1±1, and 1±3 beats/min, respectively. In the BAF312 treatment arm and throughout the 14-day study, no bradycardia was observed in any dose group tested **(** **Figure 5, D** **)**; 24-hr mean change in heart rate in the 3, 10, and 30 mg/kg dose groups were −2±2, −2±3, and −2±4 beats/min, respectively.

## Discussion

In the present study using differentially-selective, and clinically-tested and efficacious S1P receptor agonists, we demonstrate that acute bradycardia is mediated by S1P_1_ receptor activation, likely through activation of I_K(Ach)_ in pacemaker cells [5], [30], whereas the activation of the S1P_3_ receptor subtype is principally responsible for sustained hypertension as observed in the present study and in human clinical trials [14]. These conclusions are based on receptor subtype-specificity data published [31] and also generated for both compounds, as well as studies in anesthetized rats (for detection of rapid bradycardia) and conscious rats (for detection of sustained hypertension) administered FTY720 and BAF312 at doses at and above those deemed efficacious in a pre-clinical models of multiple sclerosis (mouse and rat experimental autoimmune encephalitis, EAE) whereby both FTY720 and BAF312 are efficacious at 0.3 mg/kg and which also correlated with reduced numbers of circulating lymphocytes and monocytes [32].

In anesthetized rats, both FTY720 and BAF312 produced bradycardia. While heart rate was dose-dependently decreased in the presence of FTY720, infusion of BAF312, with selectivity only for S1P_1/5_ receptors, elicited immediate decreases in heart rate during the 0.5 mg/kg (low-dose) infusion. Thereafter, decreases in heart rate were attenuated despite continued infusion and escalating plasma concentrations of the compound. A similar effect was noted for acute decreases in dP/dt in the presence of BAF312 whereby values were not maintained in the 5.0 mg/kg dose group *(please see supplementary material)*. The reduced effect on heart rate and dP/dt at high doses was likely due to ligand-receptor internalization in the presence of high concentrations of BAF312 [13] whereas complete receptor internalization was not apparent, based on the pharmacodynamic profile, in the presence of FTY720. This may be explained by the lower plasma concentrations of FTY720 achieved in anesthetized rats since the timing of the process has been shown to be highly dependent on FTY720 concentrations [9], [33]. Other S1P_1_ agonists, such as SEW2871, also elicit S1P_1_ receptor internalization [34] and results of the present study and others [13] suggest a similar phenomenon was observed in the presence of BAF312 that may help explain the transient nature of the bradycardic response in the present study in rats and in patients [6], [13].

At high enough oral doses of FTY720 (10 mg/kg/d) in rats, acute bradycardia was observed. Indeed, when the effect of the compound was monitored for 72 hours after a single oral dose, heart rate was decreased on day 1 but not thereafter despite prolonged plasma concentrations of the compound and despite sustained increases in blood pressure. The effect of FTY720 during repeated administration was tested at doses below that shown to elicit acute bradycardia. In those studies no bradycardia was observed and in fact values were increased in the 0.3 mg/kg dose group although the reason for this increase is not clear and the same effect was not observed at higher doses of the compound. More importantly, in those studies we demonstrate that FTY720 elicits dose-dependent hypertension after repeated administration at doses below those necessary to elicit bradycardia and consistent with hypertension observed clinically with FTY720 [14], [35].

Sustained hypertension in telemetry rats in the presence of FTY720 that possesses activity at S1P_3_ receptors, but not in the presence of BAF312 that lacks S1P_3_ activity, is consistent with clinical observations in patients [14], [35], [36]. These results, in concert with the receptor subtype selectivity data generated for both compounds and data from clinical trials, therefore demonstrate the importance of the S1P_3_ receptor subtype in the development of hypertension both in rats and in patients administered FTY720. Interestingly, data from a subset of telemetry animals in the present study suggest that rats administered FTY720 had a reduction in urinary sodium excretion *(unpublished preliminary observation)*, a finding consistent with a study by Tawadrous et al. [19] who demonstrated in rats treated with FTY720 (5 mg/kg/d) that the compound elicited reductions in 24-hr sodium excretion values and the fractional excretion of sodium. Whether changes in renal sodium handling were responsible for hypertension in patients or could be attributed to S1P_3_ activity of the compound is not clear.

Of note, while FTY720 elicited hypertension in telemetry rats after repeated administration, acute increases in blood pressure were not observed during an escalating i.v. infusion in anesthetized rats. In fact, blood pressure fell dramatically in that model due to the immediate and marked decrease in heart rate, and presumably cardiac output, as opposed to any direct effect on the systemic vasculature. In addition, the drop in blood pressure during i.v. infusion was exacerbated by the limited ability of the anesthetized rat to elicit a sympathetic reflexive response secondary to vasodilation [37].

Although results from the present study using two differentially selective and clinically-tested S1P agonists suggest that bradycardia in rats is mediated by S1P_1_ receptor activation, some pre-clinical studies have suggested that bradycardia may be elicited by S1P_3_ receptor stimulation and this has led to a high degree of controversy in the field and speculated differences between rodent and man. However, many of these hypotheses were proposed within SAR-driven publications with limited biological data or context. Saha et al. [38] comment that a compound, referred to as **18**, with reported selectivity for human S1P_1_ over S1P_3_, administered as an oral dose to telemetered conscious rats had no effect on heart rate, thereby implying that S1P_3_ activity is responsible for bradycardia in rodents. However, in that study heart rate was assessed only in a repeat-dosing paradigm and it has been demonstrated that bradycardia observed after stimulation of these receptors is transient in both animals and patients [3] and therefore may not have been expected after repeated administration of the compound. In another study Hale et al. [11] suggested that when compounds were synthesized with decreased S1P_3_ affinity, higher doses were required to elicit similar changes in heart rate in anesthetized rats vs. those with greater S1P_3_ affinity. Without disclosure of the hemodynamic data, nor the pharmacokinetic properties of the compounds tested in the model, it is not possible to adequately corroborate these claims. A separate study [39] also suggested S1P_3_-mediated bradycardia in rats after synthesis of differentially-selective S1P agonists due to correlation in heart rate and S1P_3_ IC_50_ values however, values correlated just as well, if not better, with activity at S1P_1_ receptors.

Lending to the hypothesis that S1P_3_ may have a role in bradycardia, Shimizu et al [10] have demonstrated that an S1P_1_ agonist, KRP-203 (which may also possess S1P_3_ antagonist properties; *unpublished observation)*, when administered as an i.v. bolus to anesthetized guinea pigs had no effect on heart rate although the highest tested dose, 0.03 mg/kg/d, was below that demonstrated to be efficacious in a disease-relevant model (0.1–1.0 mg/kg/d). However, in a separate study KRP-203 was administered to Wistar rats and while the compound had no effect on heart rate at 0.03 mg/kg, it elicited acute bradycardia (to ∼150 beats/min below baseline) at 0.1 and 0.3 mg/kg. These results clearly implicate an S1P_1_-dependent mechanism [40]. In support of this hypothesis, Murakami et al. [41] demonstrated that although the S1P_3_ antagonist, TY-52156, could partially inhibit FTY720-induced bradycardia *in vivo*, the effect of FTY720 in the presence of TY-52156 was still markedly attenuated *vs.* vehicle controls (40–50 beats/min) suggesting another S1P receptor subtype, likely S1P_1_, was responsible for the majority of the bradycardia observed in the study.

Finally, Forrest et al. [42] demonstrated that a compound, referred to as **A**, with 10-fold S1P_1_ selectivity over S1P_3_, elicited decreases in heart rate in conscious rats during i.v. infusion. However, in mice reductions in heart rate were observed only in wild type mice administered **A** or S1P, but not in S1P_3_ KO animals, thereby implicating an S1P_3_ dependent mechanism. While these data are convincing in genetically-modified mice, it may be a phenomenon limited to mouse since results of the present study in rat, and clinical studies in humans both implicate the S1P_1_ receptor subtype as the primary driver of acute bradycardia *in vivo*. Moreover, results of the present study and data reported in humans implicate the S1P_3_ receptor subtype is the primary mediator of hypertension and suggest that S1P receptor agonists devoid of activity at S1P_3_ would not be expected to elicit hypertension clinically.

*Limitations of the Study:* one cannot rule out the possibility that agonist activity at S1P_5_ is responsible for bradycardia since both FTY720 and BAF312 maintain activity at this receptor subtype. However, this possibility seems less likely since the S1P_5_ receptor has not been shown to play a role in coupling to sino-atrial nodal pacemaker activity. In addition, one cannot rule out the possibility that FTY720 agonist activity at S1P_4_ is responsible for hypertension observed in the present study since it has been suggested that this receptor subtype plays a role in vasoconstriction in the pulmonary circulation [43]. While results from a subset of animals in the present study suggest that FTY720 reduced the urinary excretion of sodium, effects on sodium balance could not be properly ascertained since accurate sodium intake values were not collected and sodium balance was also not determined in the presence of BAF312. Nevertheless, results from a separate published study in rats did suggest that FTY720 modulated the fractional excretion of sodium when administered over 28 days [19].

## Supporting Information

Figure S1 **Effect of BAF312 (0.5, 1.5, 5.0 mg/kg/20-min solubilized in a 100% PEG-400 vehicle) on left ventricular dP/dt_max_ in anesthetized rats.** Values are shown at the end of the 0.5, 1.5 and 5.0 mg/kg doses and at an equivalent timepoint in the vehicle control group. Values were statistically reduced at the end of the 1.5 mg/kg i.v. infusion, but not the 5.0 mg/kg i.v. infusion, an effect likely due to ligand-receptor internalization in the presence of high concentrations of BAF312.
(TIF)

Figure S2 **Brain, muscle, and plasma concentrations of FTY720 (A) and FTY720-P (B) after a single oral dose of FTY720 (3.0 or 10 mg/kg).** A blood sample or tissues were harvested from satellite rats at 4, 8, 24, and 72-hrs post-dose. No compound was detectable in the CSF.
(TIF)
